# Audio-Based Drone Detection and Identification Using Deep Learning Techniques with Dataset Enhancement through Generative Adversarial Networks †

**DOI:** 10.3390/s21154953

**Published:** 2021-07-21

**Authors:** Sara Al-Emadi, Abdulla Al-Ali, Abdulaziz Al-Ali

**Affiliations:** 1 Department of Computer Science and Engineering, College of Engineering, Qatar University, Doha 2713, Qatar; abdulla.alali@qu.edu.qa; 2 KINDI Center for Computing Research, College of Engineering, Qatar University, Doha 2713, Qatar; a.alali@qu.edu.qa

**Keywords:** drone, UAV, machine learning, deep learning, Convolutional Neural Network CNN, Recurrent Neural Network RNN, Convolutional Recurrent Neural Network CRNN, Generative Adversarial Networks GAN, acoustic fingerprinting, drone audio dataset, artificial intelligence, drone detection, drone identification

## Abstract

Drones are becoming increasingly popular not only for recreational purposes but in day-to-day applications in engineering, medicine, logistics, security and others. In addition to their useful applications, an alarming concern in regard to the physical infrastructure security, safety and privacy has arisen due to the potential of their use in malicious activities. To address this problem, we propose a novel solution that automates the drone detection and identification processes using a drone’s acoustic features with different deep learning algorithms. However, the lack of acoustic drone datasets hinders the ability to implement an effective solution. In this paper, we aim to fill this gap by introducing a hybrid drone acoustic dataset composed of recorded drone audio clips and artificially generated drone audio samples using a state-of-the-art deep learning technique known as the Generative Adversarial Network. Furthermore, we examine the effectiveness of using drone audio with different deep learning algorithms, namely, the Convolutional Neural Network, the Recurrent Neural Network and the Convolutional Recurrent Neural Network in drone detection and identification. Moreover, we investigate the impact of our proposed hybrid dataset in drone detection. Our findings prove the advantage of using deep learning techniques for drone detection and identification while confirming our hypothesis on the benefits of using the Generative Adversarial Networks to generate real-like drone audio clips with an aim of enhancing the detection of new and unfamiliar drones.

## 1. Introduction

In recent years, drones, also known as Unmanned Aerial Vehicles (UAVs), have become significantly popular due to the rapid technical enhancements in both their hardware, by equipping them with cameras and audio recording technologies, as well as their software, by providing the support of autonomous flying and human tracking capabilities. Initially, drones were mainly used for cinematography and recreational purposes; however, their usage has been extended to automate day-to-day operations such as vegetation monitoring [1], various wildfire mapping applications [2], precision agriculture [3] and flying over dangerous and out-of-reach areas for search and rescue missions [4]. In addition to their useful applications, their use in malicious activities to invade privacy, security and safety regulations has been increasing alarmingly. In recent events, a number of drone attacks on Gatwick airport led to the closure of the airport for a few days in an attempt to detect and manually impede the drones’ malicious missions [5]. The closure affected thousands of passengers and implied a significantly high financial cost [6]. Another incident was reported in which an explosive-equipped drone was hovering over a great crowd in a formal occasion in Venezuela, targeting a high profile personnel and the general public. In this incident, the drone dropped a number of attached explosives randomly which, consequently, injured civilians at the scene [7]. Furthermore, UAV attacks could have a negative global impact, such as the recent UAVs attacks on the Khurais oilfield and the processing plant at Abqaiq, both operated by Aramco of Saudi Arabia, causing large fires that halted their operation. This attack led to a decrease of 5.7 million barrels in crude oil production which contributed to an increase of 15% in the price of crude oil globally [8,9].

In addition to the safety issues associated with drones mentioned above, drones are also being utilized to violate security measures, as witnessed in an incident where smugglers flew drones with illegal drugs and cell phones over prison facilities [10]. Moreover, the drone violations extend to participate in disrupting sports events by flying illegally over football stadiums [11].

Similarly, privacy concerns arose with the malicious use of drones as reported in multiple incidents, where drones were used to spy and record videos and audio clips of people in their private properties [12].

**Figure 1:**
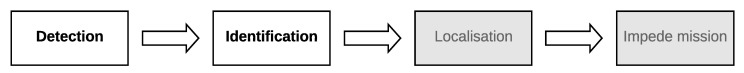
Proposed Anti-drone system.

Hence, in order to secure physical premises against malicious drone attacks, an effective approach is to design an anti-drone system that is composed of multiple stages as illustrated in Figure 1. In the first stage, the presence of a drone within a restricted area is detected. Next, the system identifies whether the drone is authorised or unauthorised through analysing its characteristics using parameters such as the drone’s type or model. Then, the system localises and tracks the malicious drone. In the final stage, the system impedes the drone’s mission using different conventional mechanisms such as shooting drones using guns, nets [13], a laser beam [14], disrupting the drone’s localization system [15] or interfering with transmission signals between the controller to hijack the drone and land it safely [16]. Various studies to secure premises against malicious drone attacks were surveyed by the authors in [17] using some of these techniques in addition to RF characteristics and wireless acoustic sensors with machine learning. Although some of these techniques were proven to be useful, each has a limitation when it comes to performing in real-life scenarios leading to an infeasibility of their deployment. Furthermore, the traditional techniques used in implementing anti-drone systems are mainly designed around the final stage of impeding the drone’s mission while being heavily dependent on manual human resources to detect and identify drones [18]. This increases the operational cost and leaves room for human errors. Therefore, this research provides a novel solution to automatically detect and identify drones using acoustic fingerprints. This solution overcomes the current limitations of the conventional anti-drone systems.

In the literature, various techniques exist to detect drones, such as:

- **Radio-Frequency (RF)** [18,19]: this technique requires a live communication of RF signals between the drone and its controller in order to detect the presence of the drone accurately. However, in scenarios where autonomous drones (preprogrammed and does not require an on-going communication) are being used in malicious activities, the RF-based system will fail to detect the presence of the drone. Furthermore, in some areas, implementing an RF system might not be applicable such as in military areas and airports. Additionally, this detection approach is subject to high RF noise emitted from other present devices in an area [20] which contributes in decreasing the Signal-to-Noise Ratio (SNR) [21]. Hence, leading to a significant deterioration of the performance of the RF-based detection system.
- **Visual Analysis**: this approach uses videos or image recognition techniques to detect drones. Although these methods have proven their effectiveness in ideal environment scenarios, their performance is heavily affected by different external factors such as weather conditions; dust, fog or rain. These recognition techniques are also susceptible to confusing similar-looking objects such as birds or kites. This is in addition to the inherit issues in visual analysis related to occlusion [20].
- **Radar**: approaches such as a GSM passive coherent location system [22] and a digital TV-based bi-static radar [23] were proposed to detect drones using radar systems. Furthermore, an attempt of an improved radar system through Doppler preprocessing was proposed in [24]. However, the experimental analysis implemented by [25] using this approach showed that the proposed solution was computationally expensive and time-consuming, which suggests that such a solution is infeasible for real-time applications. Although radars are highly effective for detecting large flying bodies, they are not efficient for detecting drones. This is due to the drones’ feature of having a low radar cross section. This is in addition to flying at low altitudes with low speed in comparison to larger aircraft [20]. Moreover, since radar systems operate at a high electromagnetic energy continuously, they might be unsuitable and illegal to operate in urban areas [20]. Furthermore, radar systems are considered expensive to deploy [21].

To address the current limitations of the drone detection systems discussed above, this study seeks to overcome the constraints of the drone detection techniques by introducing an autonomous system that, in addition to *detecting*, is able to *identify* drones based on their acoustic signatures using different deep learning techniques, namely, the Convolutional Neural Network (CNN), the Recurrent Neural Network (RNN) and the Convolutional Recurrent Neural Network (CRNN), such that no human intervention is needed. However, the following two challenges are faced by researchers in the field of drone audio analysis:

1. Lack of large acoustic drone datasets which are needed to train the deep learning algorithms effectively.
2. Most drone datasets only cover a few types of drone. Hence, not covering all types and models of drones available weakens the detection process and makes it more vulnerable to unfamiliar drone types.

To overcome these obstacles, we utilize the Generative Adversarial Network (GAN) [26], a state-of-art deep learning technique for artificial data generation to generate a large artificial drone acoustic dataset with the aim of improving the drone presence detection. Furthermore, we aim through this work to:

- Evaluate the effectiveness of the selected deep learning algorithms in drone detection and identification based on specific evaluation metrics such as accuracy, F1 score, precision and recall, while providing the computational time required to train and test the models proposed.
- Examine the validity and efficacy of combining an artificially generated dataset with a recorded drone audio dataset in enhancing the drone detection process through a comparison with only recorded drone dataset.
- Provide an open-source drone audio dataset with recorded and artificial drone audio to be further utilized by the research community in order to fulfill the shortage of drone training dataset for deep learning models.

The rest of this paper is organised as follows. Section 2 explores the literature and the state-of-art solutions, followed by Section 3, which describes the proposed framework, datasets and the neural networks architectures used in this research. Section 4 discusses the setup of the different experiments carried out through this work. In Section 5, the experimental results of the drone detection and identification approaches are presented and analysed. Finally, the conclusion is presented in Section 6.

## 2. Related Work

Several researchers focused their studies on drone detection using audio characteristics. A research was undertaken by the authors in [27], in which a new methodology through using digital signal processing (DSP) to detect the presence of drones in an area was proposed. Similarly, in the study conducted by the authors in [28], a new technique of drone detection was implemented by combining DSP with Machine Learning algorithms such as the Support Vector Machine (SVM) algorithm. It was reported by the authors the effectiveness of using SVM in drone detection which have yielded high accuracy, yet the research was limited to explicit background sounds. Moreover, SVM requires manual extraction and optimization of hand-crafted features to fine tune the model, which is an additional step to the classification solution. From this perspective, deep learning models have the capability to surpass these shortcomings and eliminate the additional steps required in the conventional machine learning algorithms by providing an end to end training of the model autonomously [29]. Following this vein, an approach was brought forward in [30] to target drone detection using DSP along with two Machine Learning algorithms, the Plotted Image Learning (PIL) and the K-Nearest Neighbor (KNN). While the algorithms demonstrated their effectiveness and detection ability, yet the overall accuracy of KNN algorithm reported was remarkably low. The authors argued that this is due to the limitation imposed by the design of the proposed solution and the fact that KNN lacks the ability of building hierarchies of internal representation which could aid in classifying similar targets. Another shortcoming is derived from the fact that PIL requires large amount of pre-stored images datasets with a consistently varying background noises to avoid biases and overfitting the noise, thus deploying such a solution in real environment is challenging.

Similarly, research undertaken by the authors in [31] surveyed the literature for some of the acoustic-based drone detection techniques, shedding light on the study conducted by [32] where a drone detection and tracking system through different DSP techniques was proposed. Although the probability of detection using the proposed solution was reported to be approximately 99% for specific ranges, it was observed that the authors have used hand-engineered values in the design of the solution. Hence, the system could fail to detect malicious drones with frequencies outside the range selected by the authors. Furthermore, the experiment was validated using one drone, indicating that if presented with a new drone, the performance of the system might be negatively affected. To avoid these limitations, in this work, we propose the use of deep learning techniques with various types of drones in order to examine and validate the robustness of the solution when faced with an unfamiliar drone.

Two of the main challenges of using an acoustic-based solution for drone detection were put forward in [31]. The first is the effect of background noise on the performance of the acoustic-based solution and the second is the availability of large amounts of diverse drone acoustic data. To address these challenges, in this research, we examine the performance of our proposed solution with the presence of various background noises in order to mimic real-life application. Furthermore, we address the second challenge by introducing a synthetic drone acoustic dataset with the aim of filling the gap in drone acoustic data.

Recent research has shown that deep learning algorithms are effective in audio applications such as speech recognition [33,34,35]. However, at present, little is known about the utilization of deep learning techniques in drone detection using drone’s acoustic features. In fact, to our best knowledge, the only study found in this field was in [36]. The authors have opted to use the Gaussian Mixture Models (GMM), RNN and CNN for this application. The authors have designed and examined the different machine and deep learning models to come to a conclusion that RNN has outperformed, in terms of F1 score, the other two algorithms.

In regard to the identification aspect of the anti-drone systems, the authors in [37] conducted a remarkable research in which they have utilized deep learning techniques for non-verbal audio identification. In their research, they studied and examined the implementation of using deep learning techniques for bird species identification which showed that such a mechanism, when used to identify bird species based on their acoustic signatures, would yield promising results. Inspired by their work, our initial work [38], which this paper is based on, aimed at designing a drone detection and identification solution using deep learning techniques, explicitly; CNN, RNN and CRNN, using recorded drone acoustic datasets [39]. We extend our study in this work to investigate what role, if any, the introduction and usage of an artificial dataset generated through GAN plays in improving the overall performance of the deep learning models as well as to verify if the artificially generated data will be good enough to fill the gap in drone audio datasets.

Recently, GANs have been used extensively in generating new images and photos of people. These images resemble a combination of features extracted from a variety of real human photos. In some cases, these photos have been modified by the GAN algorithm, through changing the hair colour or adding accessories to the human photo for example, to produce new real-like human photos [40]. Similarly, in [41], the authors introduced a new method of generating drums and piano-like audio clips using GAN models through two methods, WaveGAN and SpecGAN. Qualitatively, they evaluated the output of both experiments through human experts, in which the listeners preferred the WaveGAN audio clips over those generated through SpecGAN. To the best of our knowledge, there has been no prior literature that implements the GAN architecture to generate drone like audio to enhance detection of drones.

## 3. Proposed Framework

In this section, we discuss our proposed solution, starting with a thorough explanation of the designed research framework in Section 3.1, followed by the description of the deep learning algorithms used throughout this work in Section 3.2, and finally we breakdown our proposed drone audio datasets which we are releasing to the public and can be found in [39,42] in Section 3.3.

### 3.1. Research Framework

**Figure 2:**
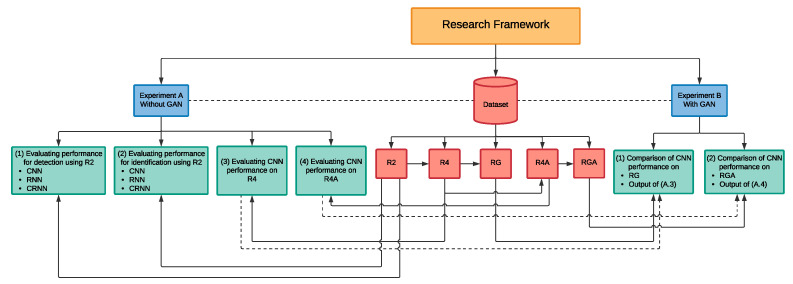
High level design of the proposed framework.

Figure 2 illustrates the design of the experiments that will be carried out throughout this research. In order to implement the proposed solution using deep learning techniques, a large amount of drone audio data were required. However, due to various reasons such as privacy, there were no public drone audio dataset available for this application in the literature as of the time of writing this paper. Hence, in our initial work published in [38], which is further demonstrated and extended in this paper under the experiments **A.1** and **A.2**, we have created our own drone audio dataset by acquiring, through audio recording using a microphone, more than 1300 audio clips of drone sounds. These clips can be found in [39]. Moreover, to mimic real life scenarios, we have used the publicly available noise datasets [43,44] to artificially augment the drone audio clips with noise. The main purpose of the artificial augmentation is to measure the feasibility of the system in a noisy environment. In addition to training the deep learning algorithm, CNN, RNN and CRNN, on the augmented sound clips, we have dedicated a portion of the dataset to include pure noise, silence and pure drone audio clips in order to ensure that the system will be able to detect and identify the drone’s sound from similar noises in an environment. Throughout this paper, we will be referring to this dataset as **R2** as it consist of audio clips of *two* drones.

We further expand, through this study, our dataset to incorporate other types of drones with the aim to consequently increase the diversification of the dataset. The new drone audio clips were collected from a variety of open-source YouTube [45] drone videos. We cleaned and preprocessed the acquired audio clips using similar techniques as those used for R2, to produce an enhanced drone audio dataset that incorporates five distinct drones, *four* of which were used in the training of the deep learning classifier and the remaining one drone is reserved for the testing phase. Hence, this dataset is referred to as **R4**. Then, by conducting experiment **A.3**, we evaluated the performance of CNN on the enhanced dataset.

By expanding the R2 dataset to R4, we aim to increase the diversification of the dataset. We speculate that the added drone types will ensure that the artificial dataset generated from the GAN model would be less biased towards a specific drone type. The dataset generated through the GAN model using R4 is referred to **RG** given that it is made up of recorded and GAN generated drone audio clips.

Hence, in the experiment **B.1**, we evaluate CNN’s performance on the RG dataset in order to establish whether adding an artificial dataset to the recorded drone audio dataset would improve the overall performance of the model by comparing the outcomes of this experiment to those found in the experiment **A.3**.

Furthermore, to evaluate the effect of altering the original drone audio clips on the classification performance, we have used pitch shifting as another data augmentation method such that it extends the R4 dataset to include the pitch shifted audio records. The enhanced dataset is referred to as **R4A**. Then, by conducting the experiment **A.4**, we evaluated the performance of CNN on this dataset.

Moreover, we hypothesised that the increase in the diversity of audio clips in R4A dataset through the integration of the pitch shifted values will allow for further generalisation and lower biases towards a specific type of drone in the artificial dataset generated from the GAN model. Hence, the dataset generated through the GAN model using R4A is referred to **RGA**.

Similar to experiment **B.1**, we investigate in the experiment **B.2** the CNN’s performance trained on the RGA dataset in order to discover whether adding a larger dataset with manually pitch-shifted audio clips would improve the data generated through GAN and hence the performance of CNN. This is achieved through a comparison analysis between the outcomes of this experiment against those found in the experiment **A.4**.

### 3.2. Deep Learning Algorithms

To implement the drone detection and identification solution, we have selected a number of well-known deep learning algorithms, namely, CNN, RNN and CRNN. The reason behind selecting each of these algorithms is expressed below:

- **CNN** is a type of deep learning algorithms that is implemented based on supervised learning to analyse, predict, categorise, and classify a given dataset. The two main features that distinguishes CNN are: (1) Patterns learnt by CNN are said to be translation invariant [46]. (2) Learning spatial hierarchies of patterns [46]. In our proposed solution, we used these unique characteristics of CNN in detecting and identifying the drones. Furthermore, we implemented the CNN architecture proposed in [47] which is built with two convolution layers followed by a linear layer and a hidden fully-connected layer.
- **RNN** is a deep learning algorithm that is used for detecting and classifying sequential and temporal data. An enhanced RNN architecture called Long Short-Term Memory (LSTM) was designed by the authors in [48] to store information through extended time sequences [49]. Taking advantage of this unique characteristic of LSTM, in this paper, the performance of RNN based on LSTM classifier in detecting whether capturing time-based dependencies improves classification performance was investigated. Moreover, we implement the RNN architecture proposed by the authors in [47] which is based on a model with a LSTM layer, output projection layer and peep-hole connections.
- **CRNN** is a hybrid architecture made up of CNN and RNN layers [50]. One of the main features of this deep learning architecture is that it combines the unique characteristics of CNN, through the utilization of the local temporal or spatial association using the CNN layers, as well as takes advantages of RNN characteristic of finding the global temporal dependencies between the different features [47]. Therefore, in this paper, we use the CRNN model proposed in [47] with convolutional layer, followed by two RNN-GRU based layers and a fully connected layer.

To implement these architectures, in this paper we have used the open-source code available in [47] to build our RNN, CNN and CRNN models. This code is an enhanced version of TensorFlow’s open-source tutorial [33]. In our implementation, the default values were used for the models’ architectures and hyperparameters per the original authors’ setup. However, it is important to note that we have modified the code to suit our application by incorporating the validation termination condition (further discussed in Section 4). By analyzing the code, the default architectures and hyperparameters proposed in [47], we inferred that the total number of trainable parameters are 69,610, 78,516 and 75,432 for CNN, RNN and CRNN models, respectively. Based on these values, we can deduce that the three deep learning models implemented in this application are similar in terms of their complexity. Furthermore, given that an essential part of designing the drone detection is to bridge the gap of the shortage of drone acoustic dataset, in this paper, we built a system based on GANs to generate new artificial drone audio clips.

The concept of GANs was first introduced by the authors in [26], where they proposed an unsupervised model build using two different types of neural networks: the Generative model *G* and the Discriminative model *D*. *D* can be any deep learning classifier. Whereas *G* is a specifically designed to generate a new set of synthetic data based on the training dataset fed to it. When it comes to training GAN, the first step is feeding a known dataset of pure drone audio and random noise as an initial input to *D*, in which it achieves a reasonable classification performance in differentiating between the drone audio samples and the noise samples. Then, *G* generates data which are initially random. As the training progresses for both models, the performance of both models improves where *G* generates better data samples based on the successful attempts of fooling *D*. In this work, we implemented the GAN model using the same architecture proposed in [41] with a slight modification to the WaveGAN’s code. The selection of WaveGAN over SpecGAN in our solution was based on the recommendation and comparative study outcome provided in [41].

### 3.3. Dataset

#### 3.3.1. R2: Recorded Drone Audio Dataset

##### Data Acquisition

To acquire the drone’s audio, we have recorded, using a microphone embedded within a smartphone, the sound generated by the drone’s propellers while flying and hovering in a quiet indoor environment within a range of distance between 1 and 20 m at different altitudes between 1 and 4 m. This enabled us to publish the dataset publicly without breaching any privacy regulations. Furthermore, we acquired a balanced number of audio clips per drone with equivalent time intervals to ensure that the audio clips will be equivalently random when fed to the algorithm to avoid any biases. This process yielded in a total audio clip of 11 min and 6 s per drone formatted in MPEG-4 audio format (m4a) with a sampling rate of 44.1 kHz and bitrate of 64 kbps.

##### Data Preprocessing

In order to prepare the audio files for deep neural networks, firstly, we reformatted the output audio clips produced from the microphone’s recording and the background noise clips by converting audio file type to WAVE, sampling rate to 16 kHz, bitrate to 16 kbps and the channel to mono to ensure consistency.

Secondly, we divided the formatted audio files into multiple short (one second) segments by specifying the time intervals at which the audio clip will be segmented, this will enable the deep learning algorithm to optimize the training of the model for real-time deployment in which the time required for the detection and identification is critical. Hence, to investigate whether the size of the audio segment affects the overall performance of the classifier, we have experimented with multiple segment sizes such as one, two and five second segments. Based on our heuristic observations, we deduced that the one second segmentation was sufficient.

**Figure 3:**
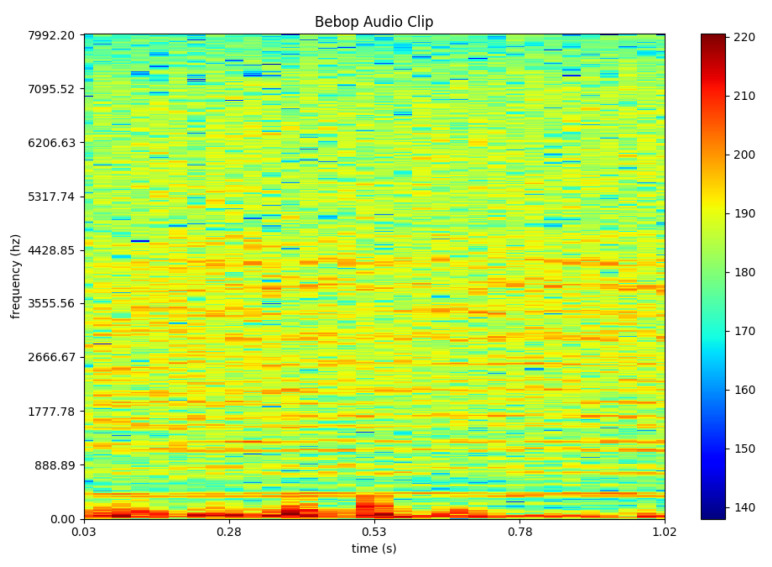
Example of drone noise in spectrogram representation © [2019] IEEE. Reprinted, with permission, from [38].

**Figure 4:**
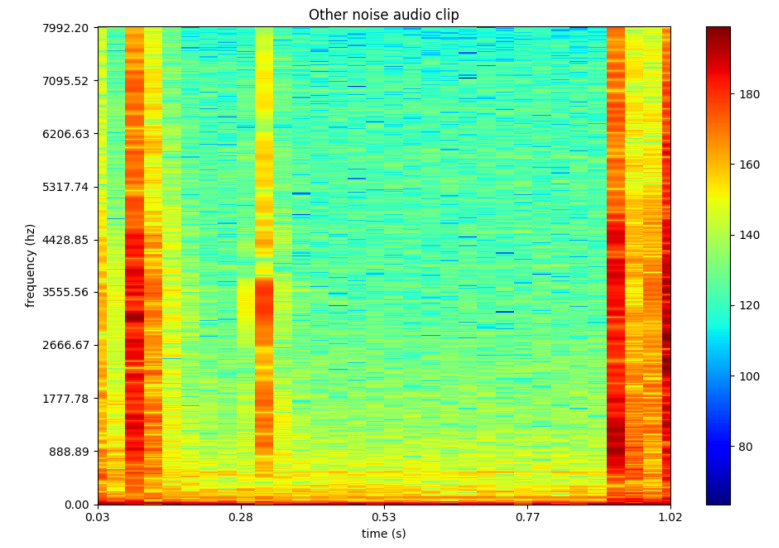
Example of other noise in spectrogram representation © [2019] IEEE. Reprinted, with permission, from [38].

One possible way to train machine learning or deep learning algorithm on audio input is by converting the audio clips into spectrograms [33]. Various features are then extracted from the generated spectrograms by the algorithm to train the deep learning models. To illustrate the outcome of this process, Figure 3 represents a one second example of a drone’s audio. Whereas Figure 4 represents an audio clip of a random noise such as a person typing.

##### Data Augmentation Using Background Noise

Since the application of the drone detection and identification could be deployed in areas with a variety of background noises, we have approached the problem by introducing a method of augmentation, in which a real-life background noise is overlapped with the drone audio without any modification on the actual audio features, such as the amplitude or the frequency of the audio clip. Particularly, we have used the background noise from the publicly available datasets [43,44]. The SNR after the background noise augmentation ranges from −32 dB to −3 dB. However, it was rather important to reformat the audio clips acquired from these datasets as discussed in Section Data Preprocessing to ensure the consistency of the audio files. Using this mechanism enabled us to mimic real-life scenarios.

##### Data Labeling

We have collected our drone acoustic data for two commercially available drones, **Bebop** and **Mambo**, manufactured by Parrot. This leaves us with **R2** dataset, which represent those *two* drones. For the identification problem, we have labelled our dataset, [39], simply as **Unknown** for other noises in an environment, **Bebop** as the first drone and **Mambo** representing the second drone. We have left four original drone audio clips for further performance analysis purposes. The distribution of audio clips acquired per label is represented in Table 1.

Similarly, for the detection aspect of this system, we have combined the data collected for both Bebop and Mambo drones as one entity and labelled them as **drone** and any other audio clip was labelled as **not a drone**.

#### 3.3.2. R4: Enhanced Recorded Drone Audio Dataset

As discussed in the introduction of Section 3.1, we expanded our drone dataset which initially consisted of two drones to incorporate other types of drones from a variety of manufacturers to be used in the drone detection experiment. The drone audio clips were collected from a variety of open-source YouTube drone videos [45] in both indoor and outdoor environments. Table 2 shows the additional drones selected along with the total number of one second clips per drone.

The selection of acquiring drone audio in two different locations, indoor and outdoor environment with general flying distances and different altitudes such as flying in an open field, was mainly to avoid the data augmentation process mentioned in Section 3.3.1 above. We manipulated the raw videos by, firstly, converting them into audio files. Secondly, we selected the relevant sections from the entire audio for this application. Finally, we divided, cleaned and preprocessed the collected drone audio clips using the same techniques as those used for R2 in Section 3.3.1 to produce the final **R4** dataset, which stands for four recorded drone audio dataset. The addition of these drones is done to increase the diversity of the R2 dataset in order to be used later on in the production of a hybrid version of the dataset using GANs.

To better understand the behaviour of the classification models on different combinations of drones, where some might be more difficult to detect than others due to their audio features’ nature, we divided our recorded drone audio dataset into five different groups referred to as ***D*** experiments, each with a different combination of drones illustrated in Table 3. In every experiment, a single *unseen* drone was not used in the training phase of the classifiers. The audio clips of the *unseen* drone was left for an exclusive testing in order to observe if the models can generalise well beyond the four drones seen during the training phase.

In each of the *D* experiments, the seen drones were grouped together and labelled as **drone** and the same noise audio clips mentioned in Section 3.3.1 above were used and labelled as **not a drone**.

In order to create different variations of the audio dataset manually, various data augmentation methods were proposed in [51] such as pitch shifting. Being inspired by their work, we selected this method as an alternative way of data generation. The semitone values used in pitch shifting the original drone audio clips were −4,−8,−12,−16, 4, 8, 12, 16. The selection of these semitone values was based on two main factors:

1. In numerous heuristic experiments, in which we ensured that the audio produced through sounded like a drone, it was observed that audio clips shifted beyond 16 semitones or below −16 semitones no longer resembled a drone to the human ears.
2. In [51], it was observed that for objects that produce a consistent audio pattern such as a drill or an air conditioner, pitch shifting with a larger range of semitone values improved the classifier performance more than those with a smaller range of semitone values. Therefore, given that the drone sound pattern is closer to those audio signals, we have made the selection of the range of the semitone values above.

Hence, the extension of R4 dataset was made through the addition of the pitch shifted drone audio which consists of an additional 420 audio records per drone, we refer to this dataset as **R4A**. The distribution of the dataset is illustrated in Table 4.

#### 3.3.3. RG: Hybrid Drone Audio Dataset

To generate the artificial drone audio dataset, we implemented a GAN model based on WaveGAN architecture described in [41]. We fed the algorithm with long pure drone audio clips which were, explicitly, recorded in an indoor environment for each of *D* experiments mentioned in Section 3.3.2. It is important to note that in every *D* experiment, the *unseen* drone was not exposed to the training of the GAN model nor in the training phase of the classifiers. After training, the GAN model generated 200 artificial drone audio clips with a duration of one second each. We have carried out human-hearing tests with a number of volunteers to test how different the GAN generated audio files are in each partition. It was concluded that there was a distinguishable difference in the sound generated for each of the *D* partitions. Those artificially generated audio clips were then combined with R4 drone dataset resulting in what we refer to as **RG**, which stands for recorded and GAN drone dataset. Table 5 shows the proportion of each in the RG dataset.

Furthermore, each set of the recorded drone clips and GAN drone clips for every *D* experiment illustrated in Table 5 were grouped together and labelled as **drone** and the same noise audio clips that were mentioned in Section 3.3.1 were used and labelled as **not a drone**.

In a similar fashion of creating the RG dataset, we have introduced the pure pitch shifted drone audio clips along with the original pure audio files to train the GAN model with the aim of increasing the diversity of the dataset fed to the GAN. Hence, the new drone-like audio clips produced through GAN were combined with R4A dataset to create **RGA** dataset. The breakdown of RGA dataset is shown in Table 6.

## 4. Experimental Setup

### 4.1. Experiments **A.1-2**: Drone Detection and Identification Using R2 Drone Audio Dataset

As already noted in Figure 2, we started our experiments by investigating the performance of the deep learning models in drone detection and identification as shown in the experiments **A.1** and **A.2** in Figure 2. This means that our initial experiment was divided into two categories, the first, **A.1**, being the binary classification experiment in which we assess the deep learning algorithms in their ability to detect whether a drone is present or not. Hence, we have defined this experiment to handle two use-cases, which are either (a) a drone was detected or (b) no drone in the area.

The second category, **A.2**, is the multi-class classification experiment, where we measured the performance of the deep learning algorithms to identify which type of drone was detected. In this experiment, there exists three distinct labels, namely **Bebop**, **Mambo** and other **Unknown** noises, to identify drones based on their type as mentioned in Section Data Labeling.

The details of the environment setup at which we deployed the algorithms, trained the models and carried out the experiments are indicated in Table 7.

In order to accomplish these objectives, we chose to evaluate and compare the different algorithms based on their accuracy, F1 score, precision and recall metrics. Additionally, we also considered the computational time (CPU time) required to train and test the model as an attribute in evaluating the performance of the models.

Furthermore, we experimented with several combinations for the ratio of training to testing datasets and we have deduced from those experiments that the variance of this ratio had minor effect on the overall performance of the model. Therefore, given that the difference is negligible, we opted to use the typical combination of 70:30.

Moreover, we have defined the distribution of the labelled data for the binary classification as well as the multi-class identification problem as presented in Table 8. The values of these parameters are defined as a result of a variety of experiments in which we have observed the model’s output by looking at the F1 Score and the accuracy in the validation phase and systematically tuned these parameters to find the optimal distribution of the dataset. It is also worth noting that the optimal learning rate found was 0.01.

### 4.2. Experiments **A.3-4** and **B.1-2**: Drone Detection Using R4, RG, R4A and RGA Drone Audio Datasets

In this experiment, we aim to determine if adding artificially generated drone audio-like data to our recorded drone data has an effect on the detection performance. More specifically, we aim to discover whether the integration of GAN generated drone audio dataset improve the performance of a deep learning classifier. Our hypothesis is that the hybrid dataset would add a generalization element which will, consequently, have a positive impact on the overall performance of the classifier.

To train the CNN classifier on the R4 dataset as mentioned in the experiment **A.3** of Figure 2 for the *seen* drones experiment, while excluding the *unseen* drone, the dataset distribution in Table 9 was used.

Table 10 shows the individual proportions of the RG dataset for each of the *D* experiments conducted in **B.1** experiment in an *unseen* drone scenario. A similar distribution was used in the experiments **A.4** and **B.2** for an *unseen* drone scenario.

In order to implement the solution proposed, an environment setup as shown in Table 11 was used in the training, validation and testing phases of the deep learning models.

## 5. Performance Evaluation

### 5.1. Experiments **A.1-2**: Drone Detection and Identification Using R2 Drone Audio Dataset

In order to ensure that every algorithm is performing at its optimum, we have carefully chosen the steps below to define the termination condition of the training phase:

1. The algorithm was executed with a very large number of training steps.
2. At an interval of 100 steps, the trained model was tested on the validation-set and the accuracy was calculated and recorded.
3. The new accuracy of the validation-set was compared against the best accuracy achieved so far.
4. If the accuracy did not improve over three successive validation tests, we tested the the trained model on the testing-set and the observed results were reported.

**Figure 5:**
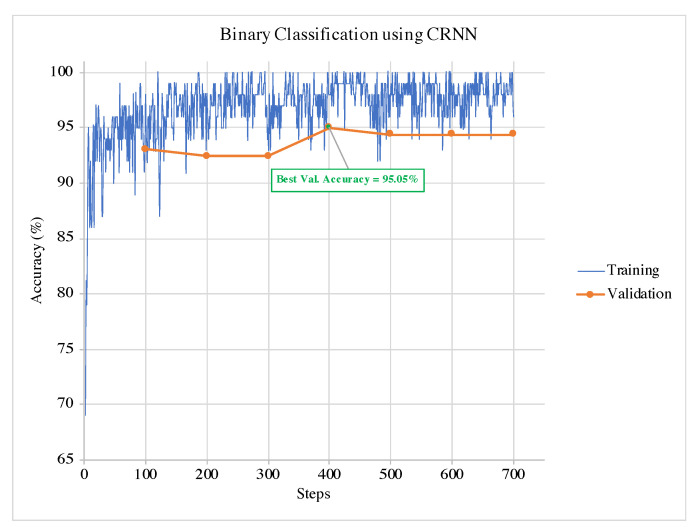
Example of the training and validation phases of CRNN for binary classification in a single run.

**Figure 6:**
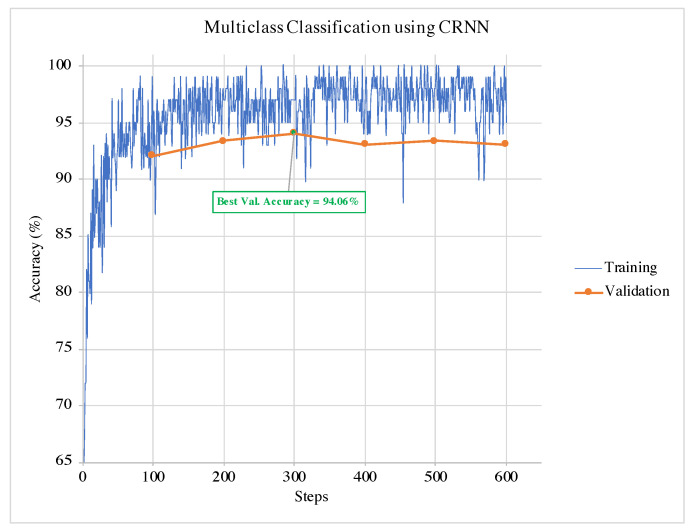
Example of the training and validation phases of CRNN for multi-class classification in a single run.

An example of training and validation of CRNN in a single run for the binary classification is illustrated in Figure 5. It can be observed from the graphs that the termination condition selects the model at the best validation accuracy. Hence, the training can terminate before the model overfits the data. The same can be observed in the example of the multi-class classification training and validation phases for and CRNN in Figure 6.

Given that the training, validation and testing datasets were shuffled randomly at the start of every execution of learning, we repeated each experiment ten times. Hence, the values discussed in Section 5.1.1 and Section 5.1.2 represent the average results of the ten runs.

#### 5.1.1. **A.1**: Drone Detection: Binary Classification Results

In this experiment, we have examined the effectiveness of our proposed system in detecting drones using their acoustic signatures. We have calculated the evaluation metrics for the three different models in addition to the corresponding standard deviation values for the 10 runs as illustrated in Table 12 below.

It can be deduced from Table 12 that CNN have outperformed RNN with a relative improvement of 21.38% in accuracy, 20.32% in F1 score, 20.32% in precision and 27.59% recall. However, the average overall training time required for CNN to yield such precise results was much higher in comparison to RNN. Whereas, it was observed that RNN had the lowest training time and overall performance among the three model. Additionally, the performance of CRNN in all evaluation criteria was better than RNN. It is important to take into consideration that the nature of RNN algorithm is best suited for sequential data. Even though, CRNN did not perform better than CNN, the difference between the performance of both models was negligible, in which CNN have shown an improvement of 1.66% in accuracy, 1.98% in F1 score, 1.21% in precision and 1.98% in recall, yet, CRNN was noticeably faster than CNN by 49.07%. This is an interesting finding because it can guide practitioners to consider the model with a lower training time without sacrificing the performance of the model.

In addition to evaluating the performance of the three different models we proposed to detect drones, we aimed to compare the output of the system with similar implementations from the literature. As of the time of writing this paper, only one source [36] was found that targets the same problem using sound *detection* approach.

The results found in detecting drones using our approach contrasts with the results found in the literature by the authors in [36]. The authors of [36] noted that RNN have achieved the best performance in comparison to CNN, whereas our results do not support their observation. In fact, we have deduced from our experimental results that CNN have outperformed RNN remarkably. There are a number of factors which might have contributed to the difference of the outcomes between the two approaches such as tuning the algorithm parameters by the authors on the testing-set directly rather than using a validation-set to serve this purpose. Moreover, the discrepancies in our findings can be attributed to the difference of the models’ architectures and design parameters such as the number of the convolutional layers used in the CNN algorithms in both applications. Due to the lack of availability of their training and testing datasets, we were not able to perform a direct comparison between the results of both approaches.

Although the results yielded from our proposed experiment do not align with those found by the authors [36], it can nevertheless be concluded that both approaches agreed on the great effectiveness of using deep learning in drone detection using acoustic features.

#### 5.1.2. **A.2**: Drone Identification: Multi-Class Classification Results

The main goal of this experiment is to examine the effectiveness of the deep learning methods in identifying drones based on their acoustic signatures. We used the evaluation metrics mentioned in Section 4.1 to examine the performance of the three models in the multi-class problem. Moreover, it is worth mentioning that the final results were calculated by taking the macro-average over all the classes in the experiment. The overall results of the evaluation metrics are presented in Table 13.

The findings that emerge from this experiment have shown that the results of both CNN and CRNN are outstanding with accuracy, precision, recall and F1 score values higher than 90%. Moreover, we observed that CNN have outstandingly outperformed RNN by an improvement of 35.78% in accuracy, 37.01% in F1 score, 33.11% in precision and 35.48% in recall. However, although RNN have shown the worst performance, it converged faster than CNN by 51.80% and than CRNN by 35.77%. In addition, it can be observed from the standard deviation values in Table 13 that RNN was the fastest to converge regardless of the difficulty of the dataset. Furthermore, it is suspected that the weak performance of RNN algorithm was due to the nature of algorithm since it is mainly based on time-dependent trend which is not the case in this experiment as the audio clips used are of a short length in which they have a constant distance with less variation over time.

**Figure 7:**
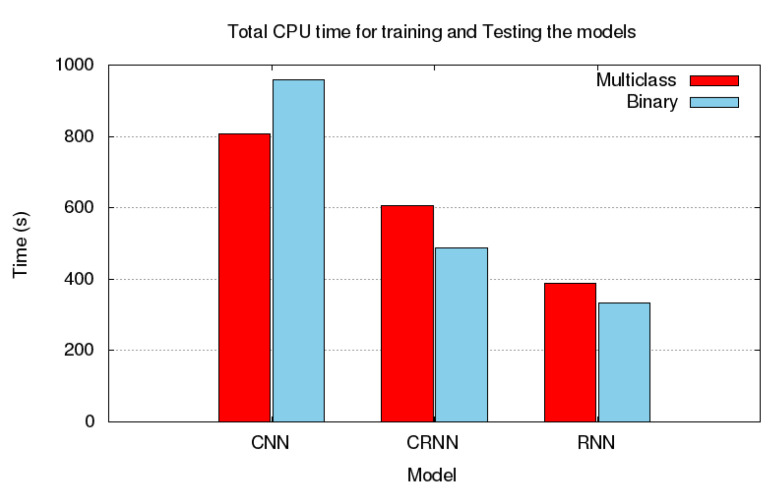
CPU time Results [38].

Moving on to the comparison between the performance of CNN and CRNN, we have observed that CNN has also performed better than CRNN by 0.72% in accuracy, 0.39% in F1 score, 0.21% in precision and 0.40% in recall. Although CNN has shown some improvement in the performance, one can deduce from the standard deviation values reported in Table 13 that the performance of CRNN is more robust in comparison to the other algorithms regardless of the data that were fed to the algorithm. Moreover, CRNN was significantly faster by 24.96% in execution time than CNN. This finding, as illustrated in Figure 7, provides a conclusive support for the results found in Section 5.1.1, since in both detection and identification aspects of the problem, it had been observed that practitioners can still utilize a model with significantly fast computational time without jeopardizing the overall performance of the model.

In addition to the results presented in Section 5.1.1 and Section 5.1.2, we have observed that the system was able to identify different drones and other noises while maintaining the precision in the evaluation matrices per label. Table 14 summarises the average performance of the 10 runs for each label in terms of F1 score for the CRNN model. The results presented below suggests that the proposed method has the ability to adjust its identification feature to accommodate more labels based on its application without sacrificing or degrading the performance per label.

Based on the findings in this experiment, where CNN have outperformed while being the most stable algorithm among the other two deep learning algorithms in drone detection using acoustic features, we will proceed with CNN as our selected deep learning algorithm for the experiments **A.3**, **A.4**, **B.1** and **B.2**.

### 5.2. Experiment **A.3** and **B.1**: Drone Detection Using R4 vs. RG Drone Audio Dataset

#### 5.2.1. **A.3**: Drone Detection Using R4 Dataset

**Figure 8:**
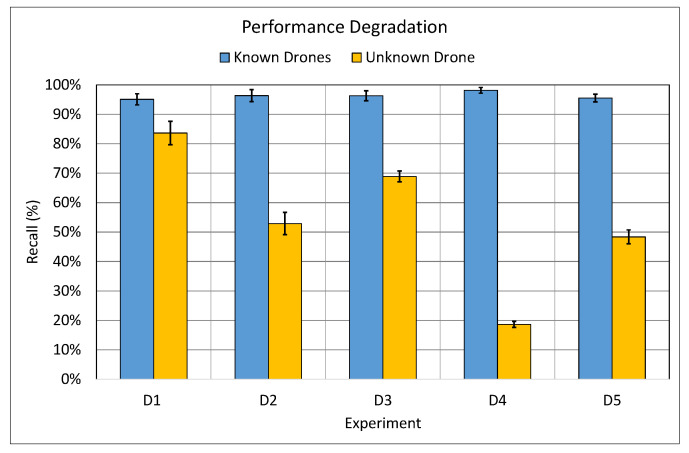
The performance, in terms of *recall*, of the average CNN models trained on the R4 drone dataset and tested on known (recorded) drone types (which the model has seen during the training phase). Whereas, the yellow bars are when tested on new and unknown types of drones.

In this experiment, ten CNN models were trained on R4 dataset then tested in every *D* partition using the testing dataset described in Section 4.2. The first testing dataset included exclusively drones types that the model has *seen* during the training phase. Whereas, the second scenario consisted of the remaining previously *unseen* drone types as the testing dataset. This type of testing was necessary in order to better understand how the model performs when faced with a completely new drone which it was not exposed to, during the training phase. For this reason, we have extended our experiments to study such behaviour. Furthermore, in order to ensure the optimal performance of each CNN model, we have followed the same four steps mentioned in Section 5.1 that defines the termination condition of training the model. The outcome of this series of experiments is illustrated in Figure 8.

The results presented in Figure 8 clearly indicate that there was a negative performance hit when the model is used to detect the presence of a drone it has never seen before (not included in the training set) which could be attributed to the lack of diversity in the training set. This behaviour can be clearly observed in the **D4** experiment where it yielded the worst classifier’s performance when met with the unknown drone, the **Mambo**. To understand the reason behind this performance hit, we have conducted human-hearing experiments with numerous volunteers. It was apparent that there was a substantial difference in the sound generated from the propellers of the **Mambo** drone to the human-ears, this difference is attributed to it being the smallest in size, in comparison to the rest drones in the training dataset of the **D4** experiment. Therefore, one can deduce that the influence of the size of the drone on the performance of the CNN model is significant and a variety of drone recordings from various drone sizes is required to enhance the performance of the classifier. Hence, it had been deduced from this experiment that the findings are consistent with our initial problem statement. Therefore, in the next experiment, **B.1**, we attempt to improve the performance of the CNN model on *unseen* drone by using the hybrid dataset, RG.

#### 5.2.2. **B.1**: Drone Detection Using RG Dataset

As observed from the experiment **A.3** previously, there is a noticeable degradation of the CNN model performance when faced with an unseen drone, hence, what we aim for by conducting this experiment is to investigate and understand whether a hybrid dataset such as RG which consists of GAN-generated drone-like audio and an actual recorded drone would have an positive impact, if any, on the overall performance of the deep learning models.

In order to guarantee the optimal performance of the CNN models, we have followed the steps mentioned in Section 5.1 to terminate the training phase of the model. It is important to note that, as of the time of writing this paper, no work was found on using GAN generated data to improve the performance of deep learning models in audio applications, hence, we put forward a novel concept to explore.

Following our initial hypothesis in which we assume that the hybrid dataset RG would improve a generalisation of our classifier, hence, it would improve the overall performance of the classifier, we divided this experiment into two sections which enable us to compare the performance of the CNN models trained on drone audio dataset without GAN, using the R4 dataset, and with GAN generated audio dataset using RG dataset.

In the experiment **A.3**, we carried out the performance evaluation experiment of the CNN models on R4 dataset to acquire the performance of the CNN model for drone detection through training and testing the proposed solution on R4 drone audio testing dataset. The outcome of this experiment will be used in evaluating the performance of the proposed hybrid RG dataset through a quantitative comparison.

Moreover, we have designed two scenarios for evaluating the CNN models on both R4 and RG datasets; the first is where the drone detected is one of the *seen* drones in which the performance of the model was examined on the same types of drones it was exposed to throughout the training phase. Whereas, the second scenario is the detection of an *unseen* drone; the drone type which was never used during the training phase. In testing the performance on an *unseen* drone, we assessed the significance of using the CNN models trained on the RG drone audio dataset to detect the new *unseen* drone described in Section Table 5 in comparison to the CNN models trained on the R4.

**Figure 9:**
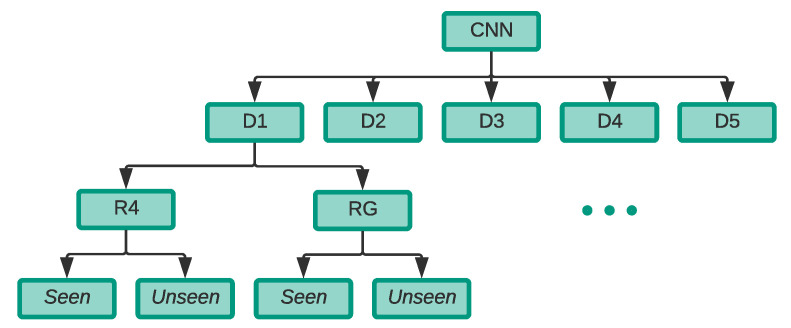
Breakdown of Experiment 2.

To achieve this aim, we have carried out the experiment as demonstrated in Figure 9 where the following steps were carefully selected for each of the five *D* partitions:

1. Train a CNN model on the R4 dataset of the *D* partition;
2. Train a CNN model on the RG dataset (composed of the selected R4 of the same *D* partition and GAN generated from the same R4 dataset of the *D* partition);
3. Test the models trained in (1) on the *seen drones* testing set from the selected *D* partition;
4. Test the models trained in (1) using the *unseen drone* testing set in the selected *D* partition;
5. Repeat (3) and (4) for the models trained in (2).

The above experiment is repeated for each of the *D* partitions ten times.

Additionally, it is important to note that for drone detection application, the most crucial evaluation metric is *recall*. As in typical intrusion detection scenario, false positive predictions are tolerated more than false negatives, where drones pass by undetected.

##### Testing on *seen* Drones

To asses whether the performance of CNN model would be improved when trained using RG dataset and tested on the *seen* drones, we conducted ten experiments for each of the *D* as mentioned in the introduction of this section. The results in Table 15 show that the CNN model trained on the RG dataset have outperformed, in terms of precision, the model trained on the R4 dataset with an increase of 0.49% in D1, 0.15% in D2, 0.10% in D3, 0.27% in D4 and 0.64% in D5. The bold values in Table 15 illustrate that, in addition to the fact that the model had better performance in terms of precision, the standard deviation is also lower.

**Figure 10:**
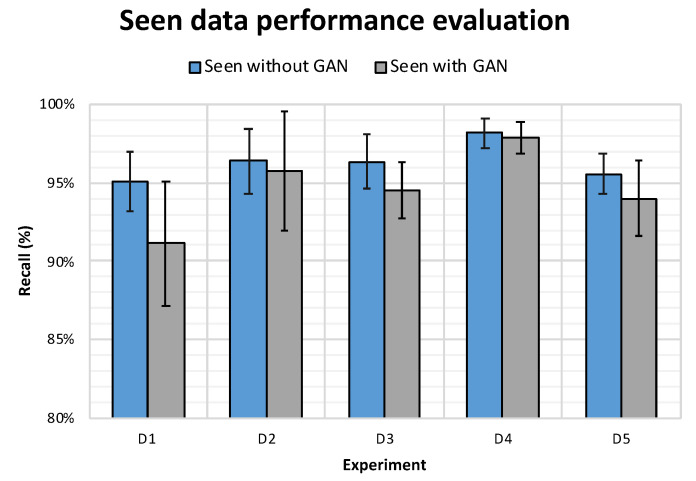
The performance of the average CNN models trained on R4 Vs. RG drone dataset and tested on *seen* drones of in terms of *recall*.

However, it can be deduced from this experiment that RG is not useful in the application where the model was already exposed to the different drone types during training given that it did not show any improvement in the other matrices, specifically recall. In fact, the models trained on RG had worse performance, in terms of recall, compared to those trained on R4 as illustrated in Figure 10. A more plausible explanation for such behaviour would be that although the performance of the CNN classifier was worst when trained on RG dataset and tested on seen drones, it is worth noting that the performance degradation was very minor.

##### Testing on *unseen* Drone

We aim through this experiment to improve the performance degradation of the classifier when it is met with an *unseen* drone which was observed in Section 5.2.1 by training the CNN models on RG dataset. To examine the model’s performance, we tested the CNN models trained on the RG dataset and R4 dataset separately using the *unseen* drone. This is in order to evaluate the generalization capabilities of the CNN models and whether the integration of artificial drone acoustic data would have any positive effect on the overall performance of the model in comparison to the observations of *seen* drone experiment in Section Testing on *seen* Drones. The results yielded from this experiment are further illustrated in Table 16.

Our study revealed that in the situation where the drone is completely new to the classifier, the average performance of the CNN model trained on the RG dataset has outperformed, in all evaluation matrices, the average performance of the model that was trained only on the R4 dataset as illustrated in Table 16. The shaded cells represent the occurrences where the model trained on the RG drone dataset has higher performance in comparison to the model trained on the R4 dataset in all five *D* experiments, whereas the bold text shows the improvement, if any, in the performance and/or standard deviation for each of the four evaluation matrices.

**Figure 11:**
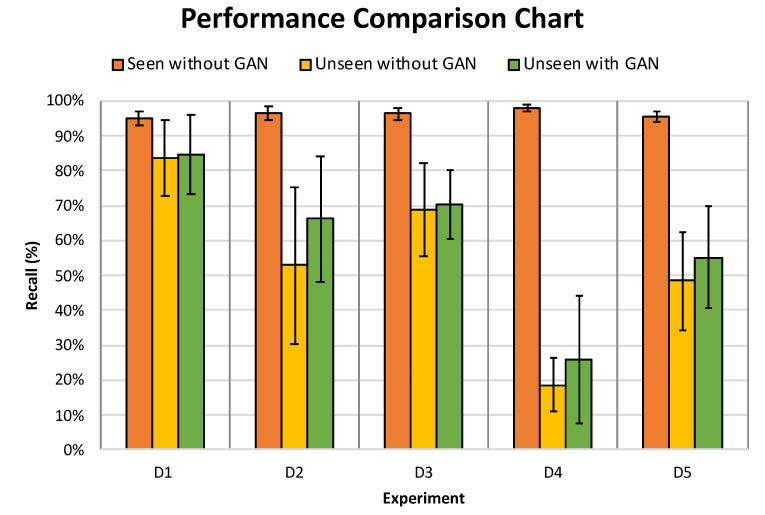
The performance of the average CNN models trained on the R4 vs. RG drone dataset and tested on *unseen* drones of in terms of *recall*.

In a similar vein, Figure 11 demonstrates the comparison between the average performance of CNN models trained on RG drone audio dataset versus the average performance of CNN model trained on R4 drone audio dataset, in terms of recall, when met with an *unseen* drone. It can be observed from the graph that there was a noticeable improvement in recall by 1.08% in D1, 25% in D2, 2.28% in D3, 38.39% in D4 and 14.16% in D5. Furthermore, this suggests that the adding GAN generated dataset to the training of a model further enhances the performance of the model in comparison to the one trained on the R4 drone dataset, particularly in recall due to the generalisation that GAN data adds to the training. Furthermore, this addition led to having a more diverse training dataset which improved the generalisation. Hence, this is a clear demonstration that using GAN in the hybrid dataset, RG, adds a significant improvement in detection of *unseen* drones in comparison to *unseen* drone detection using the recorded drone data, R4. Thus, the benefits of training CNN model on RG and using it in the *unseen* scenario outweigh the costs in the seen scenario.

This interesting finding confirms our hypothesis that the integration of the artificially generated dataset through GAN with an actual drone audio dataset not only fills the gap of drone audio shortage but it also boosts the generalization of the trained classifier for the cases of detecting completely new and *unseen* drones.

From Table 16, it is worth noting that the model trained on **D1** distribution had the best performance of around 90% in F1 score among the other experiments. This suggests that if the model was trained on DJI Phantom 4, 3DR Solo, Mambo and AR Drone, testing it on **Bebop** becomes a simple classification problem to the CNN classifier. A further explanation to this performance is that the **Bebop** drone is of a similar physical size to the majority of the drones used in the training phase. The inverse of this performance was observed in **D4** experiment, where **Mambo** drone was used in testing the performance of the model. The experiment revealed that the CNN model had the weakest performance in terms of recall, F1 score and accuracy as discussed in Section 5.2.1. Hence, one can conclude that the influence of the size of the drone on the performance of the CNN model is indisputable and a variety of drone recordings from various drone sizes is needed to further enhance the GAN model.

Concluding this section, we can say that in applications were it is expected to detect explicit types of drones that are available to train the model on, using a recorded dataset with those types of drones without GAN would be sufficient. However, in applications where detection of any type of drone is required, a hybrid dataset with GAN would be highly effective.

### 5.3. Experiment **A.4** and **B.2**: Enhanced Drone Detection Using R4A vs. RGA Drone Audio Dataset

In this experiment, we aim to evaluate the performance of the classifier on R4A and RGA datasets. In order to achieve this, we seek through this experiment to answer three main question: (1) How does the addition of synthetic audio augmentation using pitch shifting, R4A, effect the performance of the classifier in detecting *unseen* drone. (2) What are the effects on the performance of the classifier in detecting an *unseen* drone when using the synthetic audio augmentation using pitch shifting in training the GAN model, RGA, to generate a more diverse dataset. (3) What is the difference in terms of performance between the classifier trained on RGA and the classifier trained on R4A in detecting an *unseen* drone.

Table 17 represents the comparison between the average performance of CNN model trained on RGA dataset versus the average performance of CNN model trained on R4A dataset when met with an *unseen* drone. The shaded cells represent the occurrences where the model trained on the RGA dataset outperformed the model trained on the R4A dataset, whereas the bold text shows the improvement, if any, in either the overall performance, the standard deviation or both.

It can be deduced that the addition of the pitch shifted audio clips enhances the performance of the classifier. Furthermore, it can be observed that in **D1** and **D4**, the addition of the pitch shifted drone audio in RGA to generate a new dataset through GAN has improved the performance of the classifier in all evaluation metrics. We speculate that this improvement could be attributed to the fact that the pitch shifted audio records help the GAN to generate a dataset that is more diverse and could be closer to the unseen drone, in this case, the Bebop and Mambo, respectively.

In **D2**, although the classifier trained on RGA did not show an improvement in terms of recall and F1 score, the classifier performance was negligibly better in terms of precision and accuracy. This could suggest that the unseen drone, DJI Phantom 4, sounded very close to the pitch shifted audio records such that the addition of the GAN generated data did not provide a significant additional diversity in the dataset and hence did not contribute towards an enhancement in the classifier’s performance.

When investigating the performance of the classifier in **D3**, one can deduce that there was a significant improvement in the performance of the classifier when trained on RGA dataset while being more stable as can be seen from the standard deviation values in all evaluation metrics apart from precision. The same findings were found for **D5**. One possible explanation for this behaviour is that the unseen drones, 3DR Solo and AR Drone, respectively, do sound different than those provided in **D3** and **D5**, respectively. Hence, the increased diversification in RGA dataset provides more generalisation to the dataset that aids in detecting of these *unseen* drones.

Finally, we conclude this experiment with the observation that the addition of GAN generated dataset enhances the performance of the classifier in the majority of the cases and in cases where no significant improvement is observed, the classifier performs in the same manner as to when GAN is not being used.

### 5.4. Further Analysis

**Figure 12:**
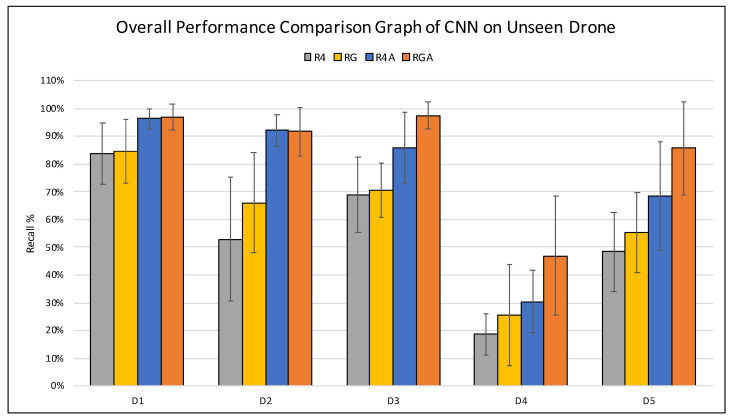
The performance of the average CNN models trained on all proposed drone datasets and tested on *unseen* drones of in terms of *recall*.

Figure 12 expresses the overall results of all the experiments conducted in this paper in terms of recall. From the graph presented in Figure 12 below, it is apparent that the manual increase in the dataset size and diversity through altering the original drone audio clips using pitch shifting contributes towards a significant increase in the classifier’s performance in detecting an *unseen* drone. This phenomena is shown in the difference of performance between R4 and R4A datasets in the graph below. Furthermore, in cases where the *unseen* drone sounds significantly different than those seen previously by the classifier, the classifier trained on the RGA dataset outperforms the others as it was observed in *D1*, *D3*, *D4* and *D5*. We speculate that this is due to the generalisation effect added by the GAN generated data. Therefore, we conclude that in this application where the drone audio datasets is scarce, integrating GAN as another method of data augmentation is useful to enhance the performance of the classifier or in worst cases perform as well as those trained on recorded and synthetically augmented using pitch shifting data. Hence, the benefits of utilising GAN for this purpose outweigh the shortcoming of not using it.

## 6. Conclusions

In this paper, we address the issue of illegal use of drones in malicious activities by proposing a novel approach that automates the drone detection and identification processes using the drone’s acoustic features with different deep learning algorithms. However, the lack of acoustic drone datasets restricts the ability to implement an effective solution using deep learning algorithms. Therefore, our work targets this gap by introducing a hybrid drone acoustic dataset, RG, composed of recorded drone audio clips and artificially generated drone audio clips using the Generative Adversarial Network (GAN). From the experiments conducted throughout this work, it was found that CNN have outperformed both RNN and CRNN in detecting and identifying drones of familiar, seen during training, types of drones.

Furthermore, when presented with seen drones, the CNN classifier trained on the recorded drone acoustic dataset, R4, outperformed the CNN classifier trained on RG dataset. However, when met with completely new drone types, the classifier was less effective and the classifier trained on RG dataset was outstandingly better. Thus, the benefits of RG dataset in the unseen scenario outweigh the costs in the seen scenario.

Additionally, when it comes to the applications where the drone audio datasets is scarce, we deduced that integrating GAN as another method of data augmentation is effective in enhancing the performance of the classifier as it will result in better or similar performance than those trained on recorded and synthetically augmented using pitch shifting data. Therefore, this suggests that the benefits of utilising GAN in such applications outweigh the shortcoming of not using it.

The proposed approach of using GANs to generate real-like drone audio clips illustrates a promising way to fulfill the gap imposed by the lack of drone acoustic dataset while also contributing to an improvement in the classifier’s performance. These findings are aimed to help the research community to use GAN generated drone audio clips along with recorded drone audio dataset, which we are releasing publicly, in various deep learning applications for further analysis.

## Figures and Tables

**Table 1:** Data per label.

Type of Drone	Records (1 s Audio Clips)			
	Original	Augmented	Total Clips for Identification	Total Clips for Detection
Bebop	331	335	666	1322
Mambo	331	335	666	

**Table 2:** Additional drones per label.

Type of Drone	Total Records (1 s Audio Clips)
3DR Solo	191
DJI Phantom 4	87
AR Drone	140

**Table 3:** Enhanced Drone Audio Dataset.

Drone Type	Experiment				
	D1	D2	D3	D4	D5
Bebop	Unseen	✓	✓	✓	✓
DJI Phantom 4	✓	Unseen	✓	✓	✓
3DR Solo	✓	✓	Unseen	✓	✓
Mambo	✓	✓	✓	Unseen	✓
AR Drone	✓	✓	✓	✓	Unseen

**Table 4:** Breakdown of R4A Dataset.

Type of Audio Clips	D1	D2	D3	D4	D5
R4 Dataset	868	1331	1248	868	1288
Pitch Shifted Audio Clips	1680	1680	1680	1680	1680
Total number of records	2548	3011	2928	2548	2968

**Table 5:** RG Drone Audio Dataset Breakdown.

Audio Type	Experiment				
	D1	D2	D3	D4	D5
Recorded Drone Clips	868	1331	1248	868	1288
GAN Drone Clips	200	200	200	200	200
Total	1068	1531	1448	1068	1488

**Table 6:** Breakdown of RGA Dataset.

Type of Audio Clips	D1	D2	D3	D4	D5
R4 Dataset	868	1331	1248	868	1288
Pitch Shifted Audio Clips	1680	1680	1680	1680	1680
GAN Generated Audio Clips	420	420	420	420	420
Total number of records	2968	3431	3348	2968	3388

**Table 7:** Experiments A.1-2 Environment Setup Details.

**Operating System**	Ubuntu 18.04-Linux
**CPU**	Intel(R) Xeon(R) x86_64 CPU E5-2695 v4 @ 2.10 GHz
**Number of CPU**	36
**Framework/APIs**	Python 2.7 and Google TensorFlow API

**Table 8:** Details on Data Distribution [38].

Criteria	Parameter
Unknown audio files	50%
**Binary Classification Problem**	
Drone audio files	50%
**Multi-class Classification Problem**	
Drone 1-Bebop	25%
Drone 2-Mambo	25%

**Table 9:** R4 Drone Audio Dataset.

Data Type	Training	Validation	Testing
Percentage	80%		20%
	70%	30%	

**Table 10:** RG Drone Audio Dataset.

Experiment	Training		Testing
	Training	Validation	
	70%	30%	
**D1**	83%		17%
**D2**	82%		18%
**D3**	81%		19%
**D4**	83%		17%
**D5**	82%		18%

**Table 11:** Experiments A.3 and B.1 Environment Setup Details.

**Operating System**	Ubuntu 18.04-Linux
**GPU**	Nvidia Titan V
**CPU**	Intel(R) Xeon(R) x8664 CPU E5-2695 v4 @2.10 GHz
**Number of CPU**	36
**Framework/APIs**	Python 3.7 and Google TensorFlow APIs

**Table 12:** Detection Results [38].

Evaluation Metric	RNN	CNN	CRNN
**CPU-Time (s)**	333.45 ± 60.90	957.33 ± 320.01	487.53 ± 178.75
**Accuracy (%)**	75.00 ± 6.60	96.38 ± 0.69	94.72 ± 1.36
**Precision (%)**	75.92 ± 10.30	96.24 ± 0.81	95.02 ± 1.14
**Recall (%)**	68.01 ± 7.59	95.60 ± 0.84	93.08 ± 1.98
**F1-score (%)**	68.38 ± 8.16	95.90 ± 0.78	93.93 ± 1.61

**Table 13:** Identification results [38].

Evaluation Metric	RNN	CNN	CRNN
**CPU-Time (s)**	389.02 ± 73.18	807.10 ± 278.09	605.67 ± 252.83
**Accuracy (%)**	57.16 ± 11.33	92.94 ± 11.89	92.22 ± 1.03
**Precision (%)**	59.64 ± 13.56	92.75 ± 1.26	92.54 ± 0.95
**Recall (%)**	57.16 ± 11.27	92.63 ± 1.32	92.23 ± 1.03
**F1-score (%)**	55.62 ± 13.53	92.63 ± 1.32	92.25 ± 1.01

**Table 14:** F1 scores per label for CRNN.

Label	Unknown	Bebop	Mambo
**F1 Score**	92.766%	93.780%	90.192%

**Table 15:** The average results of the testing on Seen Drones for every *D* experiments.

Experiment		Performance of the CNN Classifier			
		Precision	Recall	F1 Score	Accuracy
**D1**	R4	0.9773 ± 0.0100	**0.9509** ± **0.0189**	**0.9638** ± **0.0113**	**0.8630** ± **0.0171**
	RG	**0.9821** ± **0.0058**	0.9111 ± 0.0401	0.9448 ± 0.0220	0.8269 ± 0.0364
**D2**	R4	0.9883 ± 0.0045	**0.9636** ± **0.0202**	**0.9756** ± **0.0094**	**0.8740** ± **0.0183**
	RG	**0.9898** ± **0.0040**	0.9575 ± 0.0378	0.9730 ± 0.0194	0.8686 ± 0.0343
**D3**	R4	0.9859 ± 0.0050	**0.9633** ± **0.0171**	**0.9744** ± **0.0085**	**0.8735** ± **0.0155**
	RG	**0.9869** ± **0.0040**	0.9450 ± 0.0182	0.9654 ± 0.0098	0.8569 ± 0.0165
**D4**	R4	0.9884 ± 0.0037	**0.9815** ± **0.0097**	**0.9849** ± **0.0044**	**0.8908** ± **0.0088**
	RG	**0.9911** ± **0.0025**	0.9782 ± 0.0102	0.9846 ± 0.0051	0.8878 ± 0.0092
**D5**	R4	0.9853 ± 0.0044	**0.9553** ± **0.0130**	**0.9700** ± **0.0058**	**0.8665** ± **0.0118**
	RG	**0.9916** ± **0.0036**	0.9401 ± 0.0236	0.9649 ± 0.0115	0.8527 ± 0.0214

**Table 16:** The average results of testing each *D* experiment on an Unseen Drone.

Experiment		Performance of the CNN Classifier			
		Precision	Recall	F1 Score	Accuracy
**D1**	R4	0.9826 ± 0.0055	0.8365 ± 0.1097	0.8996 ± 0.0685	0.7600 ± 0.0996
	RG	**0.9861** ± **0.0025**	**0.8455** ± 0.1150	**0.9059** ± 0.0722	**0.7682** ± 0.1045
**D2**	R4	0.9790 ± 0.0252	0.5287 ± 0.2239	0.6602 ± 0.1962	0.4792 ± 0.2030
	RG	**0.9892** ± **0.0124**	**0.6609** ± **0.1807**	**0.7767** ± **0.1462**	**0.5990** ± **0.1638**
**D3**	R4	0.9774 ± 0.0106	0.6890 ± 0.1347	0.8011 ± 0.0915	0.6237 ± 0.1219
	RG	**0.9836** ± **0.0082**	**0.7047** ± **0.0987**	**0.8172** ± **0.0701**	**0.6379** ± **0.0893**
**D4**	R4	0.9363 ± 0.0128	0.1860 ± 0.0747	0.3045 ± 0.0960	0.1690 ± 0.0679
	RG	**0.9552** ± 0.0198	**0.2574** ± 0.1824	**0.3791** ± 0.1912	**0.2338** ± 0.1657
**D5**	R4	0.9752 ± 0.0128	0.4836 ± 0.1421	0.6358 ± 0.1128	0.4396 ± 0.1292
	RG	**0.9882** ± **0.0048**	**0.5521** ± 0.1442	**0.6980** ± 0.1131	**0.5019** ± 0.1311

**Table 17:** The average results of testing each *D* experiment on Unseen Drone using R4A and RGA datasets.

Experiment		Performance of the CNN Classifier			
		Precision	Recall	F1 Score	Accuracy
**D1**	R4A	0.9874 ± 0.0041	0.9644 ± 0.0362	0.9753 ± 0.0175	0.9563 ± 0.0300
	RGA	**0.9886** ± **0.0028**	**0.9695** ± 0.0453	**0.9784** ± 0.0239	**0.9621** ± 0.0400
**D2**	R4A	0.9916 ± 0.0108	0.9207 ± 0.0559	0.9538 ± 0.0292	0.9208 ± 0.0487
	RGA	**0.9989** ± **0.0034**	0.9161 ± 0.0878	0.9534 ± 0.0493	**0.9229** ± 0.0788
**D3**	R4A	0.9891 ± 0.0066	0.8581 ± 0.1268	0.9136 ± 0.0730	0.8626 ± 0.1112
	RGA	0.9783 ± 0.0147	**0.9738** ± **0.0488**	**0.9753** ± **0.0260**	**0.9564** ± **0.0436**
**D4**	R4A	0.9609 ± 0.0148	0.3032 ± 0.1121	0.4508 ± 0.1234	0.3568 ± 0.1011
	RGA	**0.0.9645** ± **0.0126**	**0.4701** ± 0.2160	**0.6034** ± 0.2031	**0.5049** ± 0.1936
**D5**	R4A	0.9883 ± 0.0065	0.6850 ± 0.1954	0.7926 ± 0.1408	0.7058 ± 0.1750
	RGA	0.9782 ± 0.0148	**0.8579** ± **0.1671**	**0.9039** ± **0.1059**	**0.8526** ± **0.1462**

## Data Availability

Publicly available datasets were analyzed in this study. This data can be found here: [39,42].
